# Lean Six Sigma methodology to improve the discharge process in a Brazilian intensive care unit

**DOI:** 10.1590/0034-7167-2022-0538

**Published:** 2023-07-10

**Authors:** Guilherme dos Santos Zimmermann, Elena Bohomol

**Affiliations:** I Hospital Alemão Oswaldo Cruz. São Paulo, São Paulo, Brazil; II Universidade Federal de São Paulo. São Paulo, São Paulo, Brazil

**Keywords:** Total Quality Management, Patient Discharge, Intensive Care Units, Workflow, Health Services Administration, Gestão da Qualidade, Melhoria de Qualidade, Administração em Saúde, Fluxo de Trabalho, Unidades de Terapia Intensiva, Gestión de la Calidad, Mejoramiento de la Calidad, Administración en Salud, Flujo de Trabajo, Unidades de Cuidados Intensivos

## Abstract

**Objectives:**

to describe the Lean Six Sigma implementation process to improve the discharge process in a Brazilian health institution’s ICU.

**Methods:**

prospective study following the Define-Measure-Analyse-Improve-Control project development method. This method consists of five phases, namely: project definition, measurement of the starting point and data collection, analysis of results, improvement in processes, and statistical control.

**Results:**

applying Lean Six Sigma methodology following the Define-Measure-Analyse-Improve-Control in the discharge process from the intensive care unit to the inpatient unit was effective in improving processes. This improvement represented a reduction in the mean patient transfer time to the inpatient unit from 189 minutes to 75 minutes, representing a 61% improvement in discharge time.

**Conclusions:**

this article demonstrates the effectiveness of applying Lean Six Sigma methodology to improve the discharge flow in a critical unit, resulting in time and waste reduction.

## RESUMO

**Objetivos:**

descrever o processo de implementação do *Lean Seis Sigma* para melhoria do processo de alta em uma unidade de terapia intensiva brasileira.

**Método:**

foi realizado um estudo prospectivo, que seguiu o método de desenvolvimento de projetos intitulado DMAIC *(Define-Measure-Analyze-Improve-Control).* Este método constituiu cinco fases, sendo elas: a definição do projeto, mensuração do ponto inicial e coleta de dados, análise dos resultados, melhoria dos processos e controle estatístico.

**Resultados:**

a aplicação da metodologia Lean Seis Sigma foi efetiva para melhoria do processo de alta da unidade de terapia intensiva para a unidade de internação. Esta melhoria representou uma redução no tempo médio de alta de 189 para 75 minutos, totalizando uma melhoria de 61%.

**Conclusões:**

este artigo demonstra a efetividade da aplicação da metodologia Lean Seis Sigma para melhoria do fluxo de alta em uma unidade crítica, possibilitando ganhos na redução de tempo e desperdícios.

## RESUMEN

**Objetivos:**

describir el proceso de implementación de Lean Six Sigma para mejorar el proceso de alta en una unidad de cuidados intensivos brasileña.

**Métodos:**

estudio prospectivo siguiendo el método de desarrollo de proyectos denominado DMAIC *(Define-Measure-Analyze-Improve-Control)*. Este método consta de cinco fases, a saber: definición del proyecto, medición del punto de partida y recolección de datos, análisis de resultados, mejora en los procesos y control estadístico.

**Resultados:**

la aplicación de la metodología Lean Six Sigma fue efectiva para mejorar el proceso de alta de la unidad de cuidados intensivos a la unidad de hospitalización. Esta mejora representó una reducción en el tiempo promedio de alta de 189 a 75 minutos, totalizando una mejora del 61%.

**Conclusiones:**

este artículo demuestra la efectividad de la aplicación de la metodología Lean Six Sigma para mejorar el flujo de descarga en una unidad crítica, lo que resulta en la reducción de tiempo y desperdicio.

## INTRODUCTION

Continuous improvement in processes or manufacturing is a strategy adopted in many organisations, including healthcare. It requires steps such as problem identification, data collection, cause analysis, planning and implementation of improvements, verification, evaluation, and control of results. These steps can be taken by using specific methodologies, such as Lean Six Sigma (LSS), that incorporate tools and continuously consider quality assurance^(1-2)^.

This methodology stems from a business philosophy and aims to reduce waste and costs, while simultaneously improving processes with rates of 3.4 defects per million opportunities. The strategy to reduce errors to this proportion is to reduce process variation to a capability of ± six standard deviations (sigma) within specified limits^(3-4)^.

To achieve this result, LSS projects suggest applications of DMAIC (Define, Measure, Analyse, Improve and Control) cycles. These cycles follow all necessary phases to improve services or products, aiming to improve work processes, quality, satisfaction and to reduce waste rates^(5)^.

Studies have demonstrated improvements in healthcare processes that applied this methodology. One of them, conducted in a medical centre in California, United States of America (USA), showed a reduction in nurses turnaround time in a paediatric care unit, which increased the time dedicated to patient and family care and increased patient satisfaction from 87% to 95%^(6)^. Another study conducted in the state of Indiana (USA) showed a 44% reduction in the cost of assembling surgical instruments, resulting in savings of approximately one and a half million dollars for the institution^(7-8)^.

The transfer of care within hospital units is a complex situation, especially when patients are transferred from the intensive care unit (ICU) to the inpatient unit (IU). The association of clinical frailty and patients’ lack of autonomy with the time of transfer may result in adverse events due to factors such as high variability in the transfer process, lack of standardisation, communication failures, and difficulties in the transition of care^(9)^. When these factors are combined, the discharge process poses risk to the patient and must be analysed from the point of view of quality, safety, financial impacts, and the experience of the patient and family. This holistic analysis allows the development and implementation of improvement actions^(10)^.

Delays in the intra-hospital transfer process can negatively impact patients and their family. For example, research has shown that 10% of patients transferred from ICUs to the ward had an adverse event in the transfer of care, and 52% of such were avoidable^(9)^. Similar to patient safety, a poorly structured discharge process can have negative financial impacts. For example, a systematic review showed four main costs related to delays in discharge: patients who occupied beds after being clinically fit for discharge, delays in admission resulting from beds that were still occupied, pressure on the nursing staff to arrange discharge, and administrative interventions associated with organisation delays^(11)^.

The LSS methodology proves to be versatile for application in several areas, including critical environments. A study conducted in a USA ICU reduced the inpatient length of stay by 24% and the mean cost of mechanical ventilation per case by 27%^(12)^. Another study, also conducted in the USA, demonstrated the effectiveness of using the LSS methodology by showing a reduction in medical transfer time to the ICU from 214 to 84 minutes^(13)^.

The Six Sigma methodology was initially introduced in Brazil only in the mid-2000s, although without Lean tools. This methodology was mainly used in the automotive, electronics, and manufacturing sectors. Since its application in the Brazilian healthcare system is still limited, there is limited research in that field to demonstrate the Lean Six Sigma effectiveness in Brazilian healthcare institutions and to expose intersections between the nursery and management fields^(7)^. Therefore, this research question is: How is the LSS implementation process to improve the discharge process in a Brazilian health institution’s ICU?

## OBJECTIVES

To describe the LSS implementation process to improve the discharge process in a Brazilian health institution’s ICU.

## METHODS

### Ethical aspects

The project was submitted and approved by the Research Ethics Committee and all research subjects agreed to participate by signing an Informed Consent Form.

### Design, period, and place of study

This is a quantitative study to evaluate the impact of an intervention aimed to improve the discharge flow of an intensive care centre using the Lean Six Sigma methodology. The study was carried out in an Adult ICU in a tertiary high complexity private hospital in the city of São Paulo, SP, Brazil, from October 2018 to May 2019. The SQUIRE 2.0 (Standards for Quality Improvement Reporting Excellence) was used as reference during the development and structuring of the study.

The ICU has seven units - five general, one neurological, and one cardiac -, totalling 44 beds. All admitted patients have a clinical and surgical treatment profile and are mostly from emergency and operating rooms.

### Population or sample, inclusion and exclusion criteria

Professionals of the ICU with experience in bedside care and leadership were invited to collaborate in the study. The researcher and the unit manager agreed to invite thirteen professionals to compose the core team of the project, consisting of seven nurses, two physiotherapists, an administrative assistant, a medical coordinator, and two nursing coordinators.

### Study protocol and analysis of results and statistics

The DMAIC project development method was used to apply the LSS methodology:

### Phase D - Define

A core working team of professionals from the unit was created. It was coordinated by the first researcher with a Green Belt background. The project scope, goals, process metrics, performance limits, and expected results were defined.

### Phase M - Measure

This phase consisted of the preparation of the operational definition, that is, the description and measurement criteria established to collect data. Information on availability time of the inpatient bed (provided by the admission to the nursing coordinator) until discharge from the ICU was collected, measured by the transfer time to the IU recorded in the hospital’s computerised system.

Process data were tabulated and analysed using the Minitab^®^ statistical software, using the mean, median, standard deviation, and sigma capability of the sample. The initial sample consisted of the total number of discharges from two general intensive care units over a three-month period (October to December 2018).

The sigma capability was determined using the Z Bench value approximation method. This value is equivalent to the standardised index of the normal curve and was obtained by the proportion of defects in the process. A 1.5 sigma detachment was added to the Z Bench value to estimate the sigma capability of the process.

### Phase A - Analyse

In this phase, we used an analysis model based on the concept of process management, in which the set of causes (x) generates effects on the output (y) of processes. Based on that, the core team used the cause-and-effect diagram tool to determine the relationships between x and y. Initially, a brainstorming session was conducted to highlight the problems encountered in the process of patient transfer from the ICU to the IU.

The traditional 6 M categorisation was used to create the cause-and-effect diagram: Measurements, Materials, Environment/Mother Nature, Method, Machine and Manpower. However, we decided to replace Manpower with Personnel, as it better describes the hospital environment. The GUT Matrix was used to implement and prioritise the possible causes identified through the analyses of three aspects: Gravity, Urgency, and Tendency. Problems are scored from 1 to 5 on each of the characteristics, and then multiplied to identify priorities^(14)^.

After prioritising the potential causes selected by the team, statistical tests were applied to confirm whether the identified problems affected the discharge process. Using the Minitab^®^ software, three hypotheses were tested for two samples. The Welch t test was used to compare two independent groups with normal means when their variances were not equal. The Minitab^®^ software report card assistant was used to determine sample power and normality pattern. A significance level (type I error probability) of < 0.05 was used.

### Phase I - Improve

The improve phase began after identifying causes statistically associated with the ICU discharge flow. It aimed to determine and implement actions and re-analyse the improved process. Another brainstorming session was conducted with the core team to propose improvements in the elements that had a negative impact on the process and to construct action plans.

### Phase C - Control

In the control phase, the aim was to standardise the improvements made in the previous phase and monitor the performance. For one month (May/2019), the individual data chart and moving range (I-MR) were prepared to monitor the mean and variation of the process.

## RESULTS

Results will be presented in sections, each of them corresponding to a phase in the DMAIC cycle.

### Phase D - Define

For this phase, the project was defined as “Lean Six Sigma to improve discharge flow” and its objective was to optimise the availability of intensive care beds after medical discharge, reduce waste, and improve operational efficiency. The first researcher and the unit manager agreed to invite thirteen professionals to form the core team of the project, consisting of seven nurses, two physiotherapists, an administrative assistant, a medical coordinator, and two nursing coordinators. The defined project scope was:

1. Project name: Lean Six Sigma for High Flow Improvement.
2. Justification: Improving ICU discharge flow refers to optimising the availability of intensive care beds after discharge. It is necessary mainly due to the high cost of maintaining the profile of patients, as keeping patients without real clinical need generates a waste of financial resources. Moreover, there may be an improvement in the unit’s renewal rate, which directly impacts revenue generation.
3. Purpose: Improve patient discharge flow in the ICU.
4. Goal: Reduce the time between availability of the ready room and the patient’s departure to 90 minutes or less.
5. Client: Hospital and patients.
6. Customer Impact: Reduced discharge time will make patients spend less time in the ICU environment, which can contribute to a better experience and reduce possible damage caused by the environment.
7. Impact on the Company: The main advantages in improving the discharge flow are the reduction of unnecessary expenses and the indirect generation of income.

### Phase M - Measure

**Figure 1:**
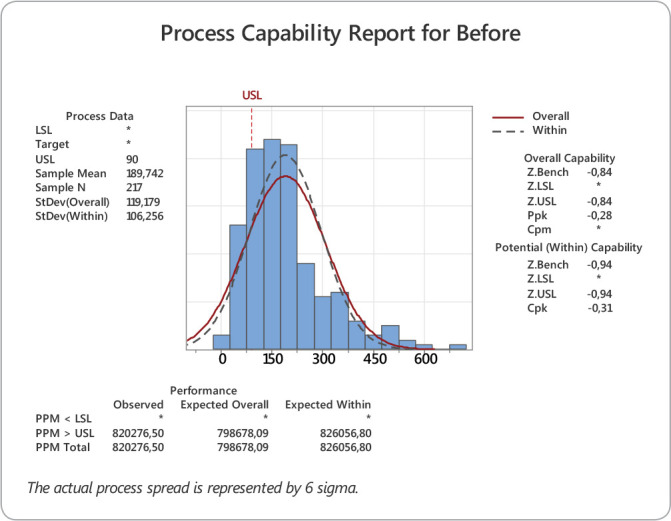
Process Capability before intervention

Capability data were collected for three months to determine the baseline. In this sample, the mean discharge time from the moment the room was ready was 189 minutes, with a standard deviation of 119.1. Capability was calculated by approximation of Z Bench, thus obtaining a sigma capability of 0.66 sigma. A histogram and a normal curve show these results graphically (Figure 1).

### Phase A - Analyse

**Figure 2:**
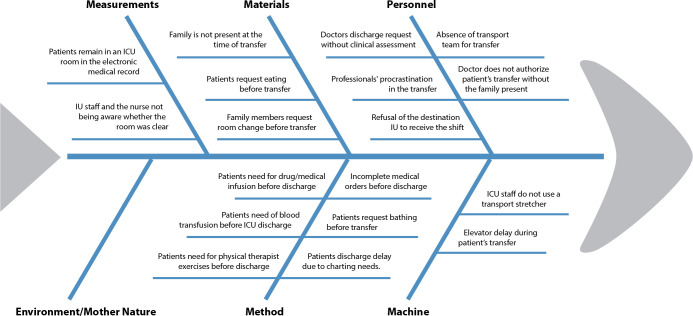
Cause and effect diagram

In Phase A, the possible causes of defects in the process were determined by the core team. The main problems due to the extended time for the transfer of patients from the ICU to the IU are shown in Figure 2.

*ICU - Intensive care unit; IU - Inpatient unit.

Another meeting with the core team was held for performing cause-and-effect analyses to identify the most important causes of delays in the intra-hospital transfer. A GUT matrix was used to identify the ten most important problems: professionals’ procrastination in the transfer (Score 100), refusal of the destination IU to receive the shift (Score 60), IU staff and the nurse not being aware whether the room was clear (Score 48), family members request room change before transfer (Score 48), elevator delay during patient’s transfer (Score 48), doctors discharge request without clinical assessment (Score 36), absence of transport team for transfer (Score 36), patients remain in an ICU room in the electronic medical record (Score 36), patients request eating before transfer (Score 27) and incomplete medical orders before discharge (Score 24).

The team selected three of the eighteen causes, namely: professionals’ procrastination in the transfer; refusal of the destination IU to receive the shift, IU staff and the nurse not being aware whether the room was clear. Thus, three hypothesis tests were designed to confirm if they significantly affect the process or not.

Cause 1: In the procrastination test, the Active Procrastination Scale was used as a reference. It uses four factors to explain the situation: preference for pressure, ability to meet deadlines, satisfaction with results, and intentional decision^(15)^. We decided to use only two factors, the preference for pressure and the ability to meet deadlines because they have a greater impact on procrastination. Group I was challenged to refer the patient to the IU within 90 minutes from the moment the room was ready, with support of the nursing coordinator and other units (Group II), without receiving any intervention. Group I had a sample mean of 74 minutes versus 237 minutes compared to Group II, with a standard deviation of 119 for the group without a goal and 28 for the group with a goal (p < 0.001).

Cause 2: In the test for evaluating the refusal of the destination IU to receive the shift, discharge samples refused in the shift transfer call and discharge samples accepted in the shift transfer call were selected. The sample of refused discharges had a sample mean of 205 minutes versus 97 minutes for the sample of accepted discharges, with a standard deviation of 102 versus and 50, respectively (p < 0.001).

Cause 3: In the test of awareness of clean rooms, one sample of discharge was collected for clean rooms (which posed no need for the nurse to periodically check the system) and another sample for rooms that were not clean (which posed a need for the nurse to periodically check the system). There was a lower mean (92 minutes) of discharges in rooms in which the fourth status were “clean” compared to rooms in which fourth status were “not clean” (133 minutes), with no statistically significant difference between mean values (p= 0.056). Although the core team believed that receiving the fourth “not clean” status of bed management would delay the patient’s departure from the ICU, this hypothesis was not confirmed because the variable was not statistically significant.

Thus, a statistically significant cause-and-effect relationship was found in two of the three problems analysed by the core team, confirming that these causes are real and not institutional paradigms.

### Phase I - Improve

In the improvement phase, the core team met to establish actions to reduce the delay in the discharge flow. After conducting a brainstorming session considering the criteria of not adding costs to the institution and establishing a shorter implementation period, the team decided for the following improvements: establishment of room release goals, revision of the reason for delays, process adjustments, implementation of discharge checklist, and on-site training.

**Figure 3:**
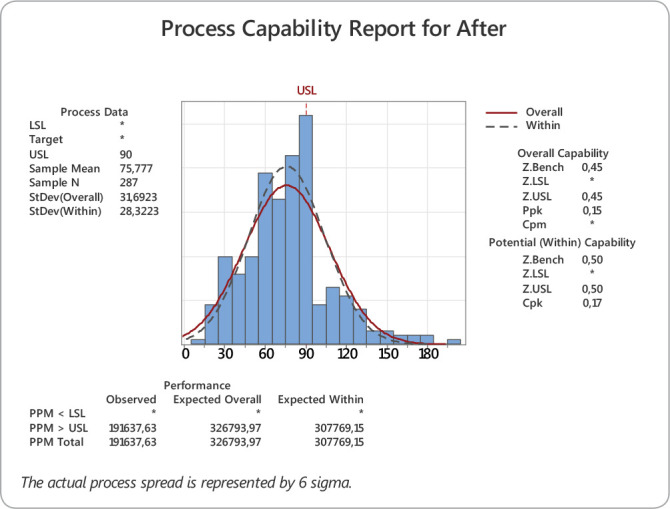
Process Capability After Intervention

After the proposal and implementation of improvements, data of discharge times were collected again. This time, all discharges from the seven ICUs (n=287) were analysed for 30 days and the sigma capability of the discharge process was calculated again, as shown in Figure 3.

In the new process, the mean time for the patient to leave the ICU for the IU was 75.7 minutes, with a standard deviation of 31.6 (maximum 200 minutes; 10 and median of 75 minutes). The new discharge process capability determined by the selected sample using the Z Bench approximation method was 1.95 sigma.

**Figure 4:**
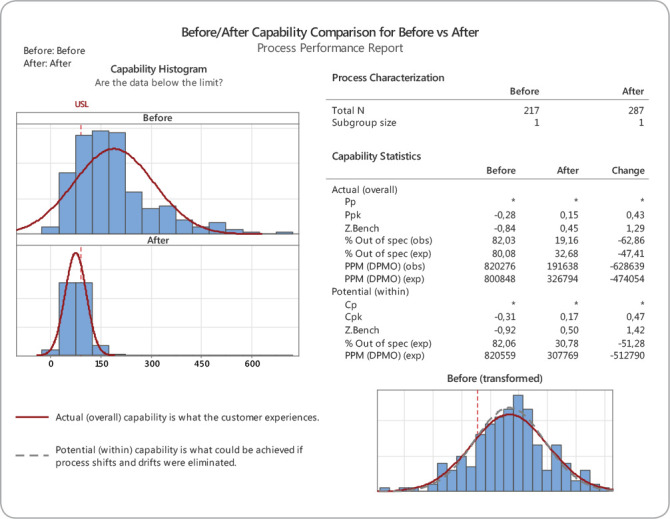
Before and After Capability Comparison

The preand post-intervention comparative analysis showed an improvement in process times, with reduction of a previous mean of 189 minutes to 75 minutes, in addition to a reduction in process variability with an improvement in sigma capability from 0.66 to 1.95 sigma, which corresponds to a 62.8% increase in discharges in up to 90 minutes. (Figure 4):

### Phase C - Control

**Figure 5:**
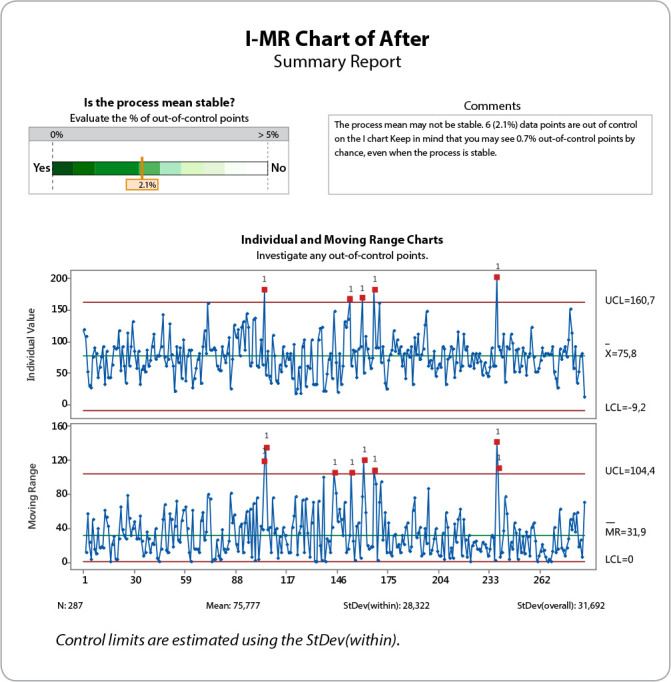
Individual data chart and moving range chart for discharge time

In the control phase, improvements were standardised and a monthly verification of the indicator and I-MR control chart was established to determine the stability of the process. The control chart created revealed the non-stability of the process with some points being outside the control limits (Figure 5).

Two out of the six observations outside the control limits were related to the absence of family members in the ICU for discharge (cases where patients could not be discharged unaccompanied to the IU), one was related to work overload and three of them had no justification, which reinforces the difficulty in dealing with notifications. As there was no stability in the process, we reinforced the need to investigate other possible causes for various other improvements not considered and to keep controlling the statistical process.

## DISCUSSION

The initial sigma capability of the discharge process was 0.4 sigma, which represents 82% of items out of specification. After setting goals, training the team, and changing the flows, the post-intervention sigma capability was 1.93 sigma, representing 19% of items out of specification. Before the intervention, the mean time taken to transfer the patients from the ICU to the IU was 189 minutes, with a standard deviation of 119. After the intervention, it was 75 minutes, with a standard deviation of 31 minutes. There was a 113-minute gain in the process, representing a 61% improvement in discharge time.

This study allowed the full method application and the development of the project using the DMAIC. The first phase consisted of listing the guidelines, the project objectives, problem identification, expected results, and goals.

This tool was useful to guide the project team and contributed to activities of the organisation. This was also found in a study conducted in an emergency service in the USA aimed at reducing the inpatient length of stay. The reported results were positive, as the tool helped the project team with problem solving during planning and subsequent phases^(16)^.

The baseline was established by determining the sigma capability of the discharge time. In this study, the capability was 0.44σ, which is out of specification of 85.44%. In a similar study conducted in Kuwait, aimed at reducing the waiting time of patients in an obstetric hospital, the sigma capability was also used as a baseline for measuring time, with initial values of 0.5σ, which corresponds to 81.48% of non-compliance^(17)^.

The choice of measuring time during the project was part of the institution’s strategic planning. This variable is inferred to be closely related to the user experience in the health service in a competitive environment that prioritises customer satisfaction. Thus, a consideration of this indicator represents a strategic decision of organisations. The 60% reduction in discharge time from ICU to IU is supported by a study that also used LSS to reduce time in an obstetric hospital, finding a 67% reduction in patient care time on weekends and 63% on weekdays, improving patient and family satisfaction^(17)^. In another study conducted in an oncology centre in Jordan in which time was measured, a 50% reduction in waiting time for medical appointments was found, improving patient care^(18)^.

Optimisations in the discharge process can bring benefits to the institution, such as the increase in bed turnover, waste reduction, and increased satisfaction. This trend is supported by the results of a study conducted in a hospital in Jordan, proposing the reduction of discharge time without increasing unit costs, which found an increase of 57% in unit costs up to the limit of 50 minutes^(19)^.

Another study conducted in a paediatric hospital in the USA aimed to reduce the request and discharge time using LSS. It had positive results in anticipating discharge requests and a 53% reduction in discharge time, with positive impacts on bed turnover despite the high hospital occupancy rate^(20)^. While all these studies established time goals in the discharge process, the research institution did not control this metric, which highlights the importance of quantitative goals for improvement of this process.

This study opted for training, encouraging care, and supporting staff to minimise procrastination, envisioning a consequent reduction in the process time. Currently, there is a trend in healthcare organisations to prioritise workers within the care process, based on the method developed by the Institute for Healthcare Improvement, the Quadruple Aim. Aiming at improving the quality of the health system, one of the dimensions of this method is the team’s wellbeing, which integrates with the others, namely: improving the patient’s experience in care, improving population health, and reducing health care costs^(21-22)^.

Thus, the application of DMAIC in its entirety brought significant gains for the institution that go beyond what was initially proposed in this study. Data show that attention was paid to bed release time throughout the organisation, discharge in IU became more predictable, proposals were made for automating processes that were human-dependent, and goals related to patient flow in the institutional remuneration program were proposed.

### Study limitations

This article demonstrates the effectiveness of applying Lean Six Sigma methodology to improve the discharge flow of a critical unit, resulting in time and waste reduction. One of the study limitations was the failure to investigate all the causes of delays. Additionally, the continuity of statistical control of the process could not be maintained due to the limited time of the research, which could have improved the sigma capability beyond the implementation of other actions. Finally, quantifying financial waste was a challenge and detailed analyses from this perspective could improve the actions that required financial investment.

### Contributions to the fields of Nursing, Health or Public Policy

This study highlights the importance of using Lean Six Sigma methodology to improve processes and quality tools in health care institutions. Also, it underlines the significance of statistical thinking and agile methods proposed by Lean Six Sigma. It contributes to the care practice through (1) encouragement to implement methods to improve processes and quality tools in healthcare institutions and (2) engagement of care professionals in methods and quality management tools, which broadens their view of the healthcare system. Finally, in management and leadership, this study develops statistical thinking and LSS agile methods and contributes to professional development in interdisciplinary methods of improvement.

## CONCLUSIONS

Applying Lean Six Sigma methodology following the DMAIC in the discharge process from the intensive care unit to the inpatient unit was effective in improving processes. Despite its origins in production, the methodology is a promising alternative for healthcare institutions, especially in patient care settings.

The initial sigma capacity of the discharge process was .44 sigma, which represents 82% of off-specification items. After goal setting, training and process adjustment, the post-intervention sigma capacity was 1.93 sigma, which accounts for 19% of off-specification items. The initial average time to transfer an ICU outpatient to the original hospital unit was 189 minutes, with a standard deviation of 119 minutes. The post-intervention average transfer time was 75 minutes, with a standard deviation of 31 minutes. The intervention warranted a total gain of time of 113 minutes, which accounts for an improvement of 61% in discharge time.
